# Keratoconus: Overview and Update on Treatment

**DOI:** 10.4103/0974-9233.61212

**Published:** 2010

**Authors:** Ladan Espandar, Jay Meyer

**Affiliations:** Department of Ophthalmology, Tulane University, New Orleans, LA, USA; 1 North Carolina University, Chapel Hill, NC USA

**Keywords:** Collagen Cross-Linking, Intacs, Keratoconus, Phakic intraocular lens

## Abstract

Keratoconus is a non-inflammatory, progressive thinning process of the cornea. It is a relatively common disorder of unknown etiology that can involve each layer of the cornea and often leads to high myopia and astigmatism. Computer- assisted corneal topography devices are valuable diagnostic tools for the diagnosis of subclinical keratoconus and for tracking the progression of the disease. The traditional conservative management of keratoconus begins with spectacle correction and contact lenses. Several newer, more invasive, treatments are currently available, especially for contact lens-intolerant patients. Intrastromal corneal ring segments can be used to reshape the abnormal cornea to improve the topographic abnormalities and visual acuity. Phakic intraocular lenses such as iris-fixated, angle-supported, posterior chamber implantable collamer and toric lenses are additional valuable options for the correction of refractive error. Corneal cross-linking is a relatively new method of stiffening the cornea to halt the progression of the disease. The future management of keratoconus will most likely incorporate multiple treatment modalities, both simultaneous and sequential, for the prevention and treatment of this disease.

## INTRODUCTION

Keratoconus is a Greek word (kerato: Cornea; konos: Cone), meaning cone-shaped protrusion of the cornea. Keratoconus is a non-inflammatory, progressive thinning of the cornea that is usually bilateral and involves the central two-thirds of the cornea.

## EPIDEMIOLOGY

Keratoconus is a relatively common disorder with a reported prevalence ranging from 50 to 230 per 100,000.^1^ Keratoconus affects all races and both sexes equally with an onset around puberty.

## ETIOLOGY

Etiology is unknown and most likely multifactorial. Several studies have reported a strong association between eye rubbing and the development of keratoconus.^2^–^6^ This association may be due to the activation of wound healing processes and signaling pathways secondary to mechanical epithelial trauma and also direct rubbing-related mechanical trauma to the keratocytes and increased hydrostatic pressure in the eye.^7^ Contact lens wear is another form of corneal microtrauma associated with keratoconus.^8^ The hereditary pattern is not predictable although the strongest evidence of genetic involvement is a high concordance rate in monozygotic twins.^9^ A positive family history has been reported in 6-8% of the cases and its prevalence in first-degree relatives is 15-67-times higher than the general population.^10^ In addition, unaffected first-degree relatives have a higher rate of abnormal topography compared with the general population.^11^ The genetic basis of keratoconus has been studied through linkage mapping and mutation analysis to reveal its molecular basis and pathogenesis. Mapping studies have identified a number of loci for autosomal-dominant inherited keratoconus: 20p11-q11,^12^,^13^ 16q22.3-q23.1,^14^ 3p14-q13,^15^ 2p24,^16^ 15q22.32-24^17^,^18^ and 5q14.3-q21.1.^19^ Other potential loci have been reported,^20^,^21^ indicating genetic heterogeneity.

## PATHOLOGY

Keratoconus can involve each layer of the cornea. The corneal epithelial cells may be enlarged and elongated.^22^ Early degeneration of basal epithelial cells can be followed by disruption of the basement membrane. This disruption results in the growth of epithelium posterior to the Bowman's layer and collagen anterior to the epithelium, forming typical Z-shaped interruptions or breaks in the Bowman's layer.^23^ Scarring of the Bowman's layer and the anterior stroma are common and present histopathologically with collagen fragmentation, fibrillation and fibroblastic activity. The stroma has normal-sized collagen fibers but low numbers of collagen lamellae, which results in stromal thinning. Endothelial cell pleomorphism and polymegathism may also be manifested. With increasing severity and duration increase, greater change and damage occurs at the base of the cone than at the apex.^24^

## CLINICAL PRESENTATION

Keratoconus patients usually present in their teenage years or twenties, complain of progressive visual blur and distortion secondary to myopia and high astigmatism. Photophobia, glare and monocular diplopia are also presenting symptoms. A scissors reflex during retinoscopy is a very early sign. Rizzutti's sign, a conical reflection on the nasal cornea when light is shone temporally, is another early sign. A Fleischer ring, or iron deposits within the epithelial layer, might be found near the base of the cone. Fine, and roughly parallel striations (Vogt lines) or stress lines of the stroma might be present. In advanced keratoconus, the corneal protrusion may cause angulation of the lower lid on downgaze, called Munson's sign. Spontaneous tears in the Descemet's membrane can result in hydrops.

## DIAGNOSTIC EVALUATION

Corneal topography is a valuable diagnostic tool for diagnosing subclinical keratoconus and for tracking the progression of the disease. Rabinowitz has suggested four quantitative videokeratographic indices for screening keratoconic patients.^25^ These indices include central corneal power >47.2 D, inferior-superior dioptric asymmetry over 1.2 D, Sim-K astigmatism >1.5 D and skewed radial axes >21°.

Maeda and Klyce developed a topographic classifier using eight indices to diagnose keratoconus.^26^ In this classifier, KPI is the keratoconus prediction index, and a value >0.23 is indicative of keratoconus. Also, the KCI% is derived using a binary decision-making tree that was input from the KPI and four other indices and a value >0 is indicative of keratoconus.

Evolution in keratoconus detection has resulted in continued refinement of indices such as the (keratometry, I-S, skew percentage, astigmatism) KISA%, described by Rabinowitz and Rasheed.^27^ The KISA% index is derived from the product of four indices: The K-value, an expression of central corneal steepening; the I-S value, an expression of the inferior-superior dioptric asymmetry; the (corneal astigmatism index), which quantifies the degree of regular corneal astigmatism (Sim K1-Sim K2); the skewed radial axis (SRAX) index, an expression of irregular astigmatism occurring in keratoconus. The following equation is used:

KISA%=(K)×(I-S)×(AST)×(SRAX)×1/3

When the KISA is100% or higher in an eye with no other pathology, the patient is very likely to have clinically detectable keratoconus. Values ranging from 60 to 100% are indicative of keratoconus suspects (with <0.5% chance of overlap with normal population), making the index useful for screening refractive surgery candidates. Patients classified as keratoconus suspects should be followed over time for signs indicating further development of keratoconus, and most likely excluded from refractive surgery.

Several topographic devices provide additional information through various features. For example, the Orbscan (Bausch and Lomb Inc., Rochester, NY, USA) provides data on anterior and posterior elevation and best-fit sphere and a corneal pachymetry map and can be used as a tool for screening keratoconus suspects. A recently introduced imaging device that provides accurate measurement of corneal power, elevation and pachymetry is the Pentacam (Oculus, Lynnwood, WA, USA). This device uses a rotating Scheimpflug camera. The Scheimpflug system determines net corneal power,^28^ elevation maps, anterior chamber depth and corneal wavefront.^29^ It is also an excellent method to detect form fruste keratoconus and keratoconus suspects by calculating the corneal thickness spatial profile and corneal volume distribution.^30^

## TREATMENT

Reduced visual acuity due to keratoconus is initially managed with spectacles. When spectacles fail to adequately correct visual acuity, contact lenses are the next option. Contact lenses often provide better vision than spectacles by masking irregular astigmatism (higher-order aberrations). For mild or moderate irregularities-soft, soft toric or custom soft toric-contact lenses can be used. Severe irregularities require rigid gas permeable (RGP) lenses in order to mask the irregular astigmatism. Various specialized RGP lenses, such as Super Cone, and Rose K, have been developed for keratoconus, with a steep central posterior curve to vault over the cone and flatter peripheral curves to approximate the more normal peripheral curvature. Some RGP lenses designed for keratoconus have high oxygen permeability and a more comfortable fit. Hybrid contact lenses are an alternative to RGP lenses. Hybrid lenses comprise a rigid center and a soft skirt. The most recent FDA- approved hybrid contact lens is SynergEyes-KC (SynergEyes Inc., Carlsbad, CA, USA), which can be used for all types of refractive errors. The lens has an RGP center and outer ring comprised of a soft lens that keeps it stable, comfortable, without toric rotation with each blink, providing consistent vision. Another alternative is the piggyback contact lens, where a soft lens is fitted to the cornea and an RGP lens is placed on top. For highly irregular corneas, gas-permeable scleral contact lenses are the last option.^31^

### Intrastromal corneal ring segments

Contact lens-intolerant patients with clear central corneas may benefit from intracorneal ring segment insertion. ICRS (Intacs; Addition Technology Inc., Sunnyvale, CA, USA) were originally developed for low-myopia correction but have now been approved for reduction of myopia and irregular astigmatism associated with keratoconus. The segments are made of polymethyl methacrylate and have a crescent-shaped arc length of 150°. The inner diameter is 6.8 mm and the outer diameter is 8.1 mm when placed in the cornea. Intacs thickness ranges from 0.25 to 0.45 mm, in 0.05 mm increments. Ring segments are inserted through mechanical and femtosecond laser-assisted corneal tunnels.

In the mechanical method, after performing a radial incision of approximately 1.8 mm in length with a calibrated diamond knife that is set at approximately 70% of the mean corneal thickness as determined by ultrasonic pachymetry, pocketing hooks are used to create corneal pockets on each side of the incision. Two semicircular dissectors are placed sequentially into the lamellar pocket to be steadily advanced by a rotational movement (counterclockwise and clockwise dissectors).^32^ In the femtosecond laser-assisted surgical procedure, the disposable glass lens of the laser system is first applanated to the cornea to fixate the eye and help maintain a precise distance from the laser head to the focal point. Then, a continuous circular stromal tunnel is created at approximately 80% of the corneal depth within 15s without further corneal manipulation.^33^ Treatment nomograms to determine optimal insert placement and thickness based on spherical equivalent, location of the cone and asymmetric astigmatism have been described.^34^

It is hypothesized that placement of ICRS induces a displacement of the local anterior surface, steepening of the peripheral cornea and a flattening of the central portion of the anterior cornea by adding extra material at the corneal midperiphery^35^ and provides biomechanical support for the thin ectaticcornea.^36^

Several studies have shown that implantation of Intacs is a safe, reversible procedure that improves objective visual outcomes and restores functional vision in most keratoconus patients.^37^–^40^ Early intervention with Intacs may also limit the development of corneal scarring associated with contact lens use. Colin *et al*. demonstrated the long-term (2-year follow-up) safety and efficacy of Intacs for the treatment of moderate to severe keratoconus with subsequent removal of Intacs in only 4.9% (four cases). They reported improvements in visual acuity and astigmatism that were stable over time and also showed significant reductions in keratometry readings at 1- and 2-year follow-up assessments compared with baseline and a slight change in K-values between 1 and 2 years. They concluded that creating a more regularly shaped corneal surface should facilitate easier contact lens fitting in keratoconus patients.^41^

After implantation of Intacs in moderate to severe keratoconus, a visually significant complication rate of 35% has also been reported,^42^ including epithelial defects, anterior and posterior perforations during channel creation, extension of incision toward the visual axis, uneven or shallow placement of implants and implant decentration. Furthermore, shallow segment depth has been associated with infectious keratitis, segment superficialization and exposure, stromal thinning, epithelial breakdown and corneal melting.^43^ Lamellar channel deposits are a common clinical finding after intrastromal corneal implants. These deposits, which primarily consist of intrastromal lipid accumulations and keratocytes, are thought to arise in response to corneal injury.^44^ Ruckhofer *et al*.^45^ reported a 74% overall incidence of intrastromal deposits. The incidence and density of deposits were directly related to the segment thickness and duration since implantation. Intrastromal deposits did not alter the optical performance of Intacs or the anatomical and physiological changes in the cornea.

Various modifications to Intacs segments have been reported. An adjustable intracorneal ring in a lamellar pocket is one reported modification. In this technique, after the formation of a closed pocket of 9 mm in diameter and 300 μm in depth within the corneal stroma, a flexible full-ring implant is inserted into the corneal pocket via a narrow incision tunnel. After insertion and evaluation of the clinical data, the implant position can be adjusted inside the pocket to achieve an optimal treatment result. An adjustment of the implant position of only 0.5 mm toward the apex of the cone may dramatically improve the surgical result. This technique enables the surgeon to choose implant diameter, implant thickness and implant position.^46^

Intacs SK, Severe Keratoconus, (Addition Technologies Inc.) is a newer design of ICRS with a smaller 6mm optical zone to correct higher grades of keratectasia with an elliptical cross-section to minimize the glare. Rodriguez *et al*. showed a clinically significant reduction in keratectasia with improvement in uncorrected visual acuity (UCVA), spherical equivalent and keratometry in patients with severe post-Lasik ectasia after placement of SK^47^

The Ferrara ring (Keravision Inc., Fremont, CA, USA) is another type of intracorneal ring that is made of polymethyl methacrylate-Perspex CQ acrylic segments. These segments also vary in thickness (0.15, 0.20, 0.30 and 0.35 mm); however, the segment cross-section is triangular, and the base for every thickness is 0.60 mm wide. The segments have 160° of arc and provide an optic zone of 5 mm. Torquetti *et al*. reported the 5-year follow-up of placement of the Ferrara ring in keratoconus patients and showed that the UCVA and the best corrected visual acuity (BCVA) improved and that there was significant post-operative corneal flattening, which remained stable.^48^

Ferrara recently reported the results of insertion of a new Ferrara ring (Ferrara Ophthalmics, Belo Horizonte, Brazil) with a 210° arc. The segments are made of acrylic Perspex CQ with an inner radius of curvature of 2.5 mm and thickness from 150 to 300 μm. The new model has been assumed to have three advantages over the conventional ring: (1) minimal astigmatic induction, (2) corneal flattening and (3) implantation of a single segment. They showed significant improvement in UCVA, BCVA and central corneal flattening, indicated by a decrease in the keratometry values.^49^

In a large retrospective, consecutive case series, Pinero *et al*. compared visual, refractive and corneal wavefront in keratoconic eyes implanted with intracorneal ring segments (Intacs or Ferrara ring) using either a mechanical or a femtosecond laser-assisted procedure for channel creation, and showed that both rings in both methods provide similar visual and refractive outcomes. Significant differences were found between the two groups for eyes implanted with Intacs for primary spherical aberration, coma and other higher-order aberrations, favoring the femtosecond group (*P <* 0.01). A significant negative correlation was found between the pre-operative corneal aberrations and the post-operative best corrected visual acuity (BSCVA) in the mechanical group (*r >* 0.63, *P ≤* 0.04).^50^ The purpose of corneal ring implantation in keratoconus patients is to reshape the cornea to improve the topographic irregularities and visual acuity. Despite the encouraging results in corneal ectatic disease after ICRS implantation, most of the patients require spectacles or contact lenses to correct residual myopia or astigmatism.

### Phakic intraocular lenses

Surgical techniques other than laser corneal refractive procedures should be considered for the correction of residual refractive error in the post-ICRS keratoconus patients. One alternative is the use of anterior or posterior phakic IOLs, including toric lenses, either alone or after implantation of ICRS.

In a prospective non-comparative interventional case series, angle-supported Phakic IOLs (ZSAL-4, Morcher GmbH) were implanted in 12 eyes with stage I-II keratoconus, myopia from – 6.5 to – 14.00 and astigmatism from – 1.00 to – 5.00. At 1 year post-operatively, spherical error was within ±1.00 D, without any significant change in astigmatism in all cases, and UCVA was 20/40 or better in all cases.^51^ The main concern with angle-supported phakic IOLs is endothelial cell loss. However, the newest generation appears to be well tolerated in normal eyes.^52^ Moshirfar *et al*. reported successful implantation of iris-supported Verisyse phakic IOL (AMO, Santa Ana, CA, USA) in two cases and found 4% endothelial cell loss at 3-months post-operatively in both cases.^52^

Alfonso *et al*. implanted the myopic Phakic posterior chamber Implantable Collamer Lens (ICL, STAAR, Monrovia, CA, USA) in 25 eyes with myopia from –3.00 to –18.00 D and astigmatism from 0.5 to 3.00 D and 12 months follow-up, and reported that the spherical equivalent refraction was within ±1.00 D of the desired refraction in all cases.^53^ Kamiya *et al*. reported implantation of a Phakic toric ICL for correction of high myopic astigmatism in two eyes with stable keratoconus (–10.00-6.00 ± 100 in case 1 and –8.00-2.75 ± 100 in case 2), and showed that after 1 year, the follow-up manifest refraction was +0.5-1.00 ± 90 in case 1 and –0.25-1.25 ± 100 in case 2, without complications.^54^

The combined implantation of intracorneal segments and IOLs is becoming more common, with a variety of lenses and techniques. The sequential implantation of Intacs and posterior chamber ICL was evaluated in three eyes, with significant improvement in UCVA and BCVA and reduced refractive spherical equivalent observed.^55^ El-Raggal *et al*. conducted a prospective evaluation of sequential implantation of Intacs and Verisyse phakic IOL in eight eyes and showed the safety, stability and effectiveness of the procedure.^56^ The simultaneous implantation of Intacs/Verisyse has also been performed with outcomes that were not significantly different compared with sequential implantation (unpublished data, personal communication, Majid Moshirfar, MD).

Progression of keratoconus leading to refractive change is a concern after implantation of any type of Phakic IOL. Ideally, Phakic IOL implantation should not be performed until refraction and keratometry are stable. Indications for Phakic IOL implantation in these cases should be BSCVA of 20/50 or better, clear central cornea, keratometric values ≤52.00 D and stable refraction (cylinder ≤3.00 D) for 2 years. If these criteria are not met, penetrating keratoplasty or collagen cross-linking (CXL) may provide better visual outcomes.

### Collagen cross-linking

The disadvantage of the previously mentioned procedures is that none adequately prevent keratoconus progression that occurs due to the underlying biomechanical corneal changes. The biomechanical characteristics of the cornea result from the collagen scaffold and collagen compound and their bonding with the collagen fibrils. The three-dimensional configuration of the collagen lamella determines the cornea's resistance. Biochemical and immunohistochemical studies of the corneal matrix proteoglycans show differences between normal and keratoconic corneas.^57^–^58^ Enzymatic alterations with an increased expression of lysosomal and proteolytic enzymes,^59^ decreased concentration of protease inhibitors,^60^ decreased thickness and modified configuration of the stromal collagen lamella^61^ have been observed. A photo-oxidative CXL technique using riboflavin and ultraviolet-A (UVA) light was developed to counteract the progressive corneal thinning, and consequently the progression, of keratoconus.

With cross-linking, additional covalent bonding between collagen molecules can be achieved, which stabilizes the collagen scaffold and changes several tissue properties.^62^

An increase in corneal stiffness and enhanced resistance against proteolytic enzymes caused by the riboflavin and UVA light were shown in enucleated pig eyes.^63^ The cross-linking effect is not distributed homogenously over the corneal depth. The stiffening effect is concentrated in the anterior 200-300 μm of the cornea due to the high absorption of UV light in this area.^64^

Raiskup-Wolf *et al*. retrospectively evaluated the long-term effect of riboflavin and UVA in progressive keratoconus with maximum follow-up of 6 years and showed that the steepening significantly decreased by 2.68 D in the first year, 2.21 D in the second year and 4.84 D in the third year, with an associated improvement in BCVA and stability. It was concluded that cross-linking is an effective therapeutic option for progressive keratoconus.^65^ Cross-linking (CXL) is discussed in greater detail in other segments of this issue.

## NEW HORIZONS

With the advent of corneal cross-linking technology to stabilize the biomechanically weakened collagen in keratoconus, the spectrum of keratoconus management now includes both the prevention and the treatment of progression of disease. It is anticipated that techniques such as CXL will become more widely available as familiarity with this procedure increases. To reshape the cornea, correct high myopia and astigmatism, and prevent progression of ectasia, the application of multiple treatment modalities such as CXL, ICRS and Phakic IOLs, either simultaneously or sequentially, will likely become more common for the treatment of all aspects of this disease.
